# Motor current and vibration monitoring dataset for various faults in an E-motor-driven centrifugal pump

**DOI:** 10.1016/j.dib.2023.109987

**Published:** 2023-12-17

**Authors:** S. Bruinsma, R.D. Geertsma, R. Loendersloot, T. Tinga

**Affiliations:** a Royal Netherlands Navy, Den Helder, The Netherlands; b Netherlands Defence Academy, Den Helder, The Netherlands; c University of Twente, Faculty of Engineering Technology, Enschede, The Netherlands

**Keywords:** Vibration analysis, Motor current signature analysis, Condition based maintenance, Bearings, Induction motor, Variable frequency drive, Fault detection, Fault identification

## Abstract

Induction motor driven pumps are a staple in many sectors of industry, and crucial equipment in naval ships. Such machines can suffer from a wide variety of issues, which may cause it to not perform its function. This can either be due to degradation of components over time, or due to incorrect installation or usage. Unexpected failure of the machine causes downtime and lowers the availability. In some cases, it can even lead to collateral damage. To prevent collateral damage and optimise the availability, many asset owners apply condition monitoring, measuring the dynamic response of the system while in operation. Two high-frequency measurement methods are widely accepted for the detection of faults in rotating machinery at an early stage: vibration measurements, and motor current and voltage measurements. These methods can also distinguish between different failure mechanisms and severities. The dataset described in this article presents experimental data of two centrifugal pumps, driven by induction motors through a variable frequency drive. Besides measurements of behaviour that is considered healthy (new bearings, well aligned), the machines have also been subjected to a variety of (simulated) faults. These faults include bearing defects, loose foot, impeller damage, stator winding short, broken rotor bar, soft foot, misalignment, unbalance, coupling degradation, cavitation and bent shaft. Most faults were implemented at multiple levels of severity for multiple motor speeds. Both vibration measurements, and current and voltage measurements were recorded for all cases. The dataset holds value for a wide range of engineers and researchers working on the development and validation of methods for damage detection, identification and diagnostics. Due to the extensive documentation of the presented data, labelling of the data is close to perfect, which makes the data particularly suitable for developing and training machine learning and other AI algorithms.

Specifications Table

**Table:** 

Subject	Engineering
Specific subject area	Vibration, voltage and current measurements for the detection of multiple faults of different severities for condition monitoring purposes.
Data format	Pre-processed
Type of data	Table
Data collection	The vibration measurements were obtained using accelerometers, with a sample rate of 20 kHz. A measurement was taken every five minutes and lasted 60 s. The data is pre-processed to .csv files per channel (5 channels total), split in columns of 12 s, containing the acceleration in g. The electrical current was obtained using one current clamp per phase and the voltage was obtained using one voltage tap per phase, both with a sample rate of 20 kHz. A measurement was taken every two minutes and lasted 15 s. For each phase, the current and voltage is stored in .csv files per channel (three current, three voltage), split in columns of 15 s, containing current in ampere (A) or voltage in volt (V).
Data source location	The data was collected at the Techport Fieldlab at Tata Steel, IJmuiden, the Netherlands.
Data accessibility	Repository name: 4TU.ResearchData Data identification number: 10.4121/2b61183e-c14f-4131–829b-cc4822c369d0 Direct URL to data: https://data.4tu.nl/ndownloader/items/2b61183e-c14f-4131–829b-cc4822c369d0/versions/1

## 1 Value of the Data

• This dataset is unique in its broad scope of different facets and extensive documentation. This dataset contains individual, reproducible fault data of two machines, using two measurement techniques for 20 different faults, most at multiple levels of severity and, for one motor, at three different running speeds. The severities are considered realistic and challenging to detect, in contrast to many ‘proof of principle’ datasets with unrealistically large faults presented in some current literature. Also, documentation of the faults and operational settings is extensive, yielding near perfect labelling of the data. Finally, a large amount of measurements representing normal behaviour is included in the dataset as a baseline.
• This data can be used to better understand the dynamic response of rotating machinery to different faults occurring in real-world systems. Engineers and researchers in the field of reliability, condition monitoring and data science can therefore benefit from this dataset.
• This dataset can aid in the development, training and/or (re-)testing of novel (AI, machine learning) methods and models in pursuit of fault detection, identification and diagnosis in electric motors and pumps. The use of this kind of data in such algorithms have already been extensively reviewed [1]. A typical example is the use of statistical parameters in the time and frequency domains like root mean square and skewness [2]. It can also serve as a benchmark dataset for comparing the performance of new and existing fault detection and identification methods, like the ones proposed by Bold and Urschel [3] or Umbrajkaar et al. [4].
• The motivation for the set-up used to generate this data is that induction motor pump set-ups are very prevalent machines in the industry, also containing failure modes (unbalance, misalignment, or bearing faults) that occur in other (rotating) equipment. Further, vibration and current/voltage monitoring are considered two of the most applied condition monitoring techniques for this type of equipment [5].

## 2 Data Description

**Fig. 1:**
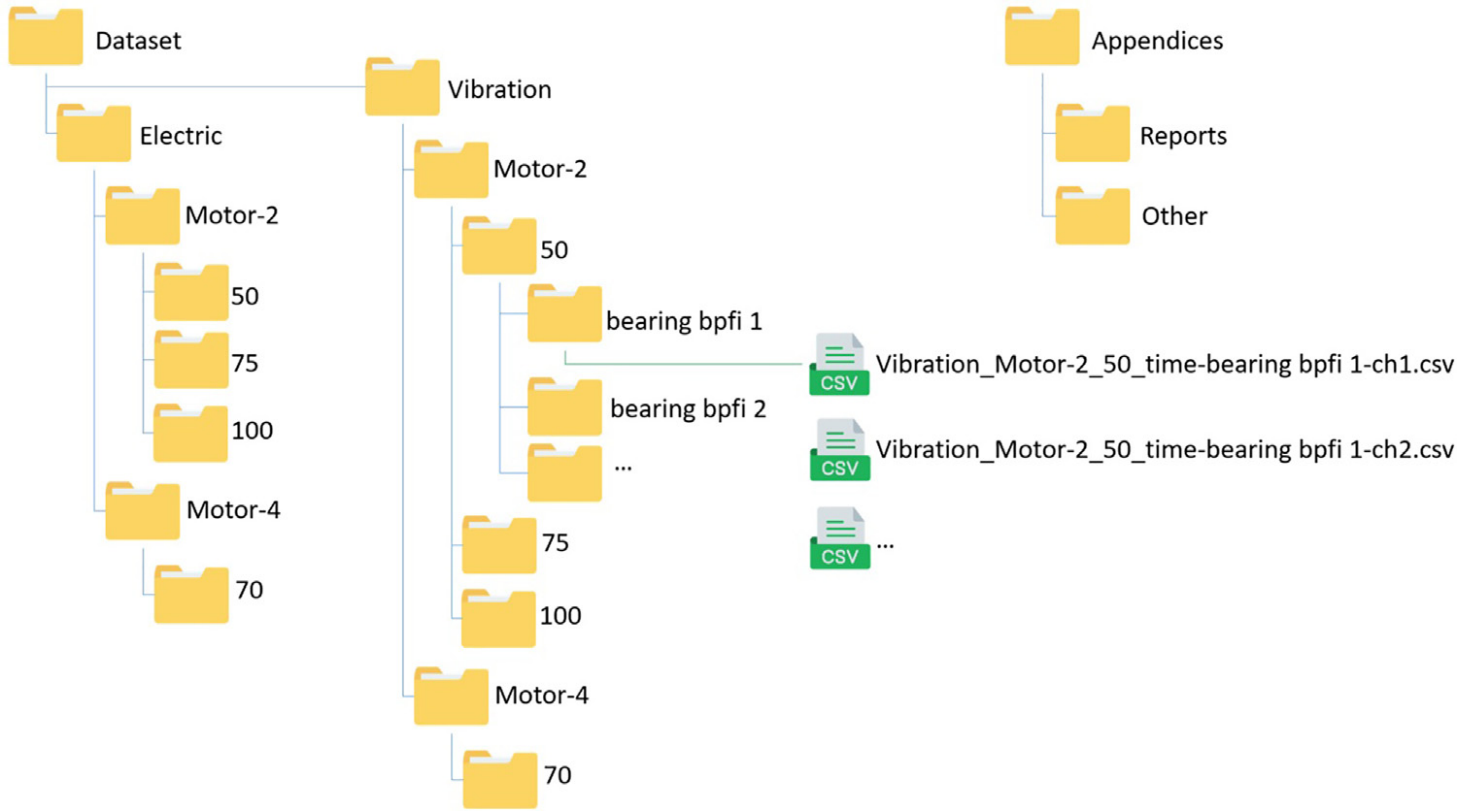
Schematic overview of file structure.

The dataset is organised in a file folder structure, schematically displayed in Fig. 1. The dataset itself is compressed in a .7z file to minimise storage requirements. The first layer splits the data between the two measurement methods, namely ‘Vibration’ and ‘Electric’. The second layer splits the data between the two set-ups over which the failures are divided, these are named ‘Motor 2’ and ‘Motor 4’. The next layer splits the data based on rotational speed of the electric motor. ‘Motor 4’ is only tested at 70% of the rated speed. ‘Motor 2’ is tested at 50, 75 and 100% of the rated speed. Finally, the data is separated over the different failure phenomena folders. In this case, the number refers to the severity level, where a higher number means a higher severity, with exception to the healthy case.

The measurements are stored in .csv files. The files themselves have the same base name as the folder they are in, specifying the measurement method, motor number, speed, fault and severity. This name is succeeded by the channel number. In case of vibration measurements, the channel specifies the location of the measurement, explained in Fig. 4 in the Measurements section. In case of current and voltage measurements, the first three channels contain the current in the separate phases, the last three channels contain the voltages in those phases, as explained in Table 3. The .csv files themselves contain column headers, the first column contains the time in seconds, starting at *t_1_* = 1/20000 s. For the vibration measurements, all other columns contain acceleration in g of the separate measurements. Every column has the same length of 240.000 values, corresponding to 12 s of measurements. For the current and voltage measurements, the columns contain phase current in ampere (A) and phase voltage in volt (V) of separate measurements. Every column has the same length of 300.000 values, corresponding to 15 s of measurements.

In the Appendices folder, there is a subfolder called reports. In this folder, the balancing and alignment reports are located. Furthermore, there is a folder containing datasheets of the electric motors and pumps used in the experiments. Finally, there is an Excel file with an overview of all measurements.

## 3 Experimental Design, Materials and Methods

The experiment has been designed such that data is generated in a structured manner for two measurement methods, four motor speeds and 11 (simulated) fault types at various severity levels. In this section, the experimental details will be presented, starting with the set-up of the motor-driven pumps and the used measurement, and data acquisition hard- and software. After that, all induced faults are documented in detail.

### 3.1 Experimental set-up

**Fig. 2:**
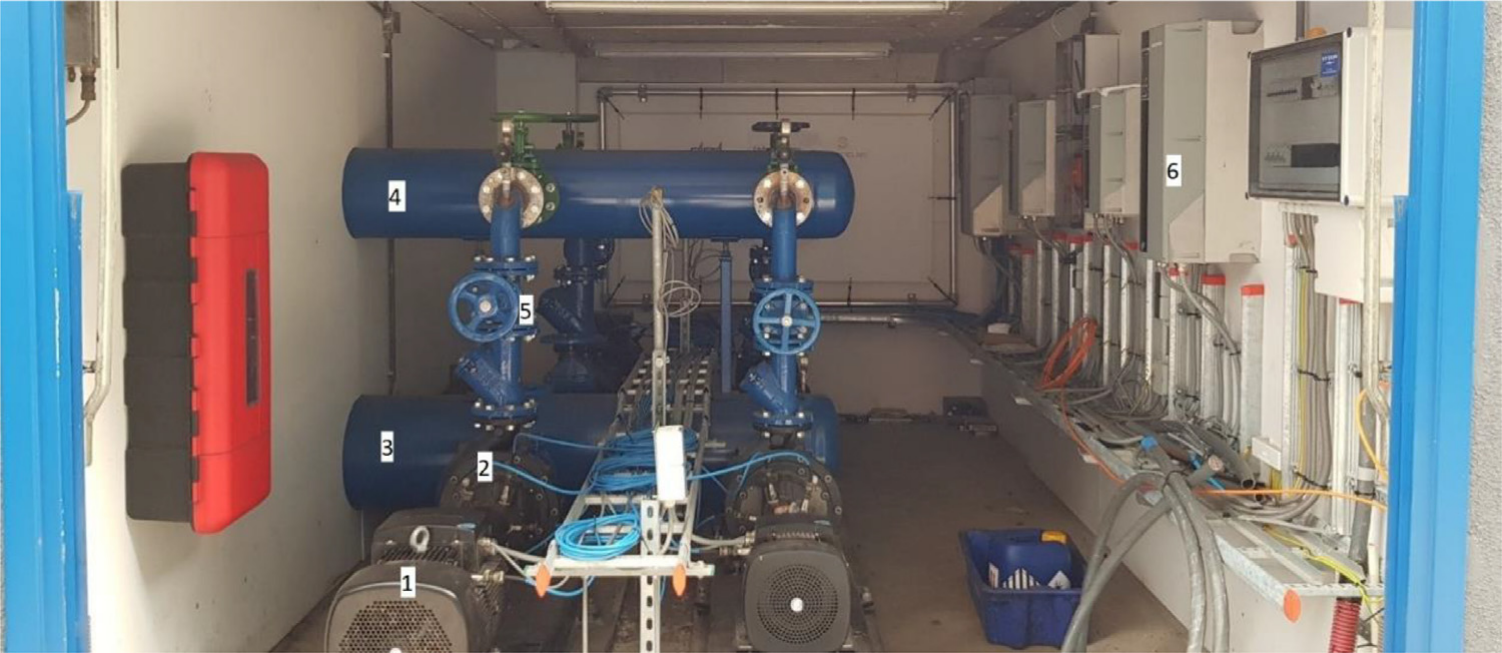
Impression of the experimental set-up with (1) Electric motor, (2) Pump, (3) main water inlet, (4) main water outlet, (5) discharge valve and (6) Variable Frequency Drive (VFD).

The complete set-up contains four centrifugal pumps, each driven by an induction motor, see Fig. 2. Only two of the set-ups were used in this experiment. Circulated fluid (fresh water) is obtained from a 60 m^3^ storage tank and transported through a system of pipes, containing valves before and after each pump to control the flow. The specific details of the e-motors and pumps are given in Tables 1 and 2, respectively.

**Table 1:** Details of used Electric motors.

Nr.	Name	Poles	U_max_(V)/I_max_(A)	kW @ RPM	Bearings
2	MG160MA4042-H3	4 pole	380–415/23.4–22.4	11 @ 1470	6309.C4
4	MG180MB2–48-F1	2 pole	380–415/43.5	22 @ 2950	6310.C4

**Table 2:** Details of used pumps.

Nr.	Name	Coupler	Flow(m^3^/h) @ RPM	Bearings
2	NK80–250/270 A2F2AE-SBQQE	Spacer	119.9 @ 1470	6308.2Z.C3.SYN
4	NK80–160/167 A2F2AE-SBQQE	Spacer	200.4 @ 2950	6306.2Z.C3.SYN

### 3.2 Measurements

**Fig. 3:**
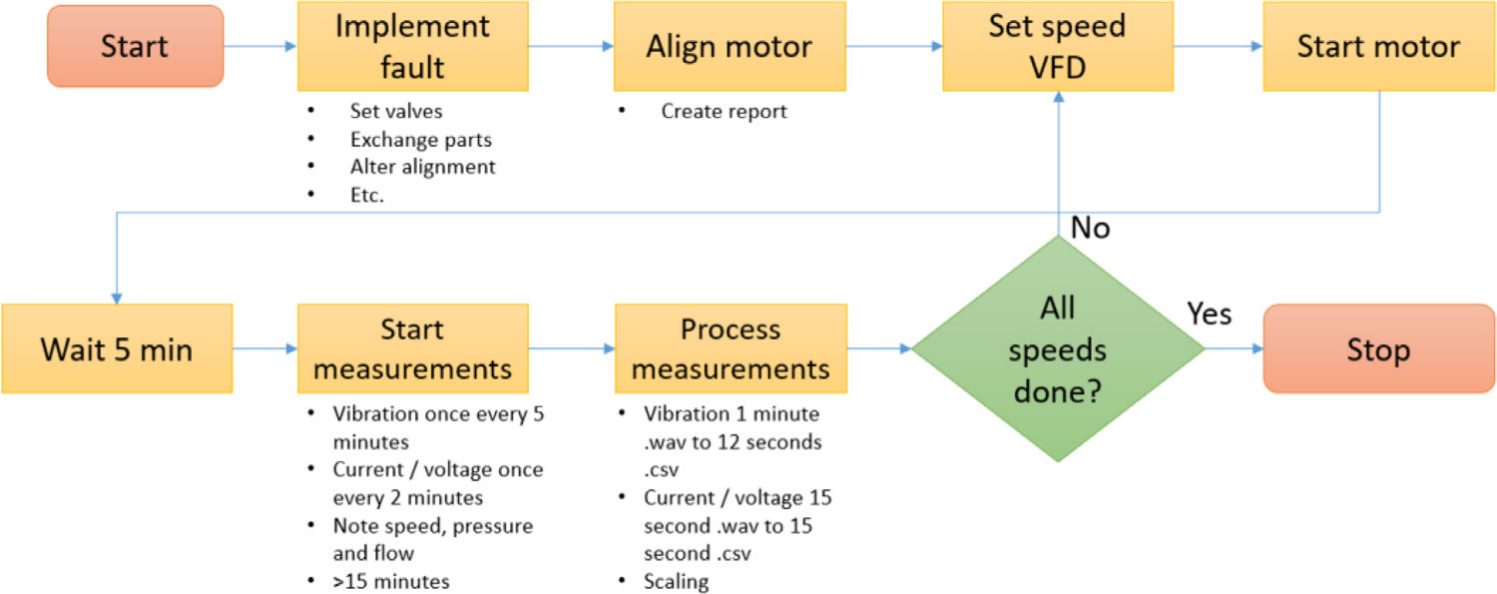
Flowchart measurement acquisition.

To acquire the measurements, the flowchart in Fig. 3 was used. Every fault was implemented individually by exchanging parts or changing settings in the set-up, like alignment or valve settings. Afterwards, the alignment of the motor with respect to the pump has been validated and corrected if necessary. Next, the variable frequency drive (VFD) was set at the correct speed before the motor was started. The set-up was given some time to reach steady operation. From this moment on, the measurements were saved automatically for a duration of at least 15 min. The data was processed per motor number, speed and measurement method. If another measurement for a different speed was required for that motor, the speed was changed and the testing route repeated. Otherwise, the motor is stopped.

**Fig. 4:**
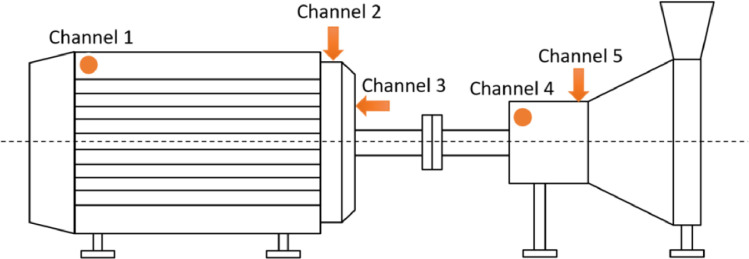
Vibration measurement locations, arrows show location and direction, dots represent direction perpendicular to the schematic drawing.

The following measurements are taken during the experiments:

Vibrations: The raw vibration measurements were taken with 5 Wilcoxon 786B-10 100 mV/g single axis accelerometers [6]. These were connected to a National Instruments Data Acquisition Unit (USB-4432). The data capture software was written by Istec International BV. The sample rate was set to 20 kHz. The low and high cut-off frequencies were set to 1 Hz and 10.000 Hz respectively, by means of a Butterworth band-pass filter of the second order. A measurement was taken every 5 min, lasting 60 s. The sensors were placed on the relevant bearing locations of the electric motor and pump, as specified in Fig. 4. The gathered data was scaled to the dimensionless range of [−1,1] in the data file, with a depth of 24 bits. This corresponds to the maximum allowable acceleration of [−50,50] g. The resulting .wav files were pre-processed to .csv files per channel with correct scaling in chunks of 12 s per column.

Current & voltage: The electrical current was measured using CR Magnetics CR3110 current clamps [7]. The voltage was measured using Wago 855 voltage taps [8]. Both were measured for all three phases, after the VFD. The data infrastructure was supplied by Samotics BV. The sample rate is set to 20 kHz, the same as for the vibration measurements. A measurement was taken every 2 min, lasting 15 s. During data collection, the first three channels contain the current in the separate phases, the last three channels contain the voltages in those phases, as explained in Table 3. The raw 24 bit .wav files were pre-processed to .csv files, separated per channel, scaled to denote current and voltage in the .csv files.

**Table 3:** Channel assignments current and voltage.

Channel	Channel 1	Channel 2	Channel 3	Channel 4	Channel 5	Channel 6
Phase	Phase 1	Phase 2	Phase 3	Phase 1	Phase 2	Phase 3
Current/voltage	Current	Current	Current	Voltage	Voltage	Voltage

Operational settings: In addition to the data collected with the two condition monitoring methods, the operational settings of the motor-pump set were also registered. These values were taken as a single snapshot during each test. This means that for each fault condition, the following quantities / settings were determined:

• Motor speed (RPM): this value was read from a Compact Instruments Ltd MiniVLS 112 [9] with an accuracy of 5 RPM.
• Fluid Flow (m^3^/h): this value was read from an UFM KATFLOW 200 [10] with an accuracy of 5 m^3^/h.
• Discharge Pressure (bar): this value was read from a generic analogue pressure gauge with an accuracy of 0.1 bar.
• Alignment reports, measured with a Prüftechnik Rotalign touch [11].
• Balancing reports, measured externally.

### 3.3 Induced faults

A range of fault types, outlined in Table 4, was introduced in the set-up, and both vibration and current measurements are collected for each of these faults. Every fault was introduced individually, and only one single fault was present at the same time. The machine was restored to a healthy state at the start of each measurement, including validation of the alignment every time. Each of the fault types will be discussed in detail below.

**Table 4:** Simulated faults.

Motor 2 (3 different speeds)			Motor 4 (1 speed)		
Main fault	Specific	Severities	Main fault	Specific	Severities
Bearing	Motor, BPFO	3 severities	Misalignment	Angular	5 severities
	Motor, BPFI	3 severities		Parallel	4 severities
	Pump, BPFI + BPFO^1^	3 severities		Combination	4 severities
	Motor, BSF	1 severity	Unbalance	Motor side	6 severities
	Motor, contaminated	1 severity		Pump side	3 severities
Loose foot	Motor	1 severity	Coupling	Various steps	4 severities
	Pump	1 severity	Cavitation	Inlet	4 severities
Impeller	Damaged blades	3 severities		Outlet	5 severities
Stator	Winding short	2 severities	Bent shaft	Rotor	1 severity
Rotor bar	Broken	1 severity			
Soft foot	Motor	2 severities			

^1^Only BPFO was intended, but BPFI turned out to be present also, see E-motor bearing damage below.

#### 3.3.1 Healthy, baseline

In order to compare all failure simulations, a normal, reference behaviour should be established. This was done by acquiring healthy data of the motor-pump set-ups. To be considered healthy in this test, the misalignment must be less than 0.05 mm, relative to perfect alignment. In between simulations of failures, this level of alignment was reacquired, to ensure a singular failure phenomenon per case. Before initiating the tests, the electric motors and pumps were fitted with new bearings and seals. After some initial tests, the first healthy measurements were acquired. The healthy data was collected at several separate moments during the whole series of experiments. These separate moments are denoted as Healthy 1, 2 and 3. These are not severity cases, but represent different moments in time.

#### 3.3.2 Stator winding short (and new motor)

**Fig. 5:**
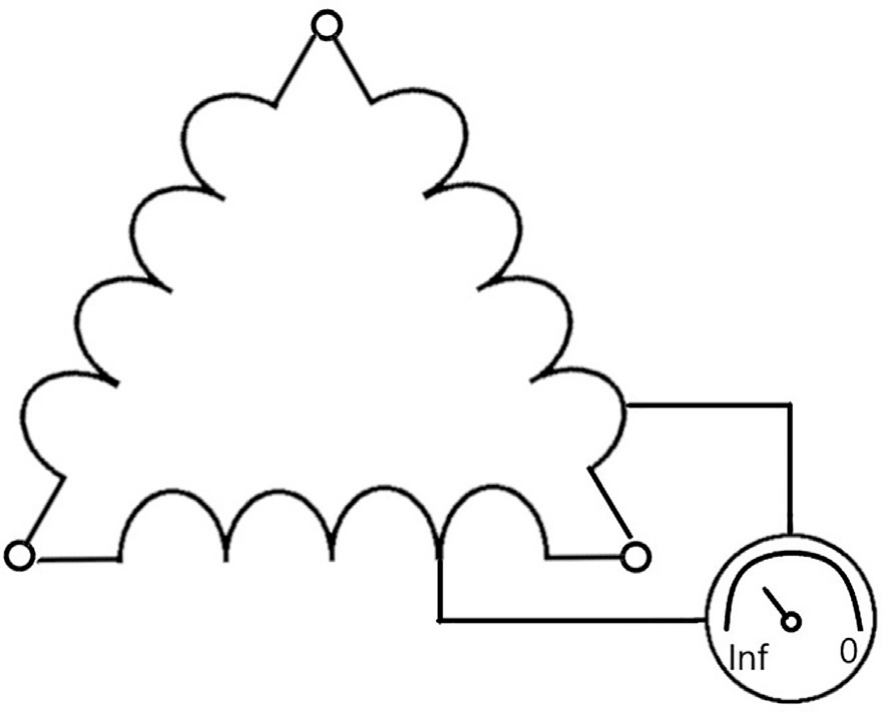
Schematic of modified motor with resistance transformer.

To simulate a stator winding short due to isolation degradation, a new electric motor was modified before the test. The modification was carried out by Facta Uitgeest BV. Two stator windings were interconnected via a transformer, simulating a phase to phase winding short. The transformer uses a potentiometer to regulate the resistance. When the potentiometer is fully closed, infinite resistance, no electrical current will run through it and the electric motor will operate normally. This state is included as “new motor” and should be considered healthy. When the potentiometer is fully open, minimal resistance, the full electrical current will flow from one winding, through the transformer, to the other winding. An analogue dial on the transformer allows for setting the magnitude of the electrical current through the transformer, see Fig. 5. This was used to simulate the two severities; approximately 25% and 50% of the phase current was by-passed through the transformer.

#### 3.3.3 Broken rotor bar

**Fig. 6:**
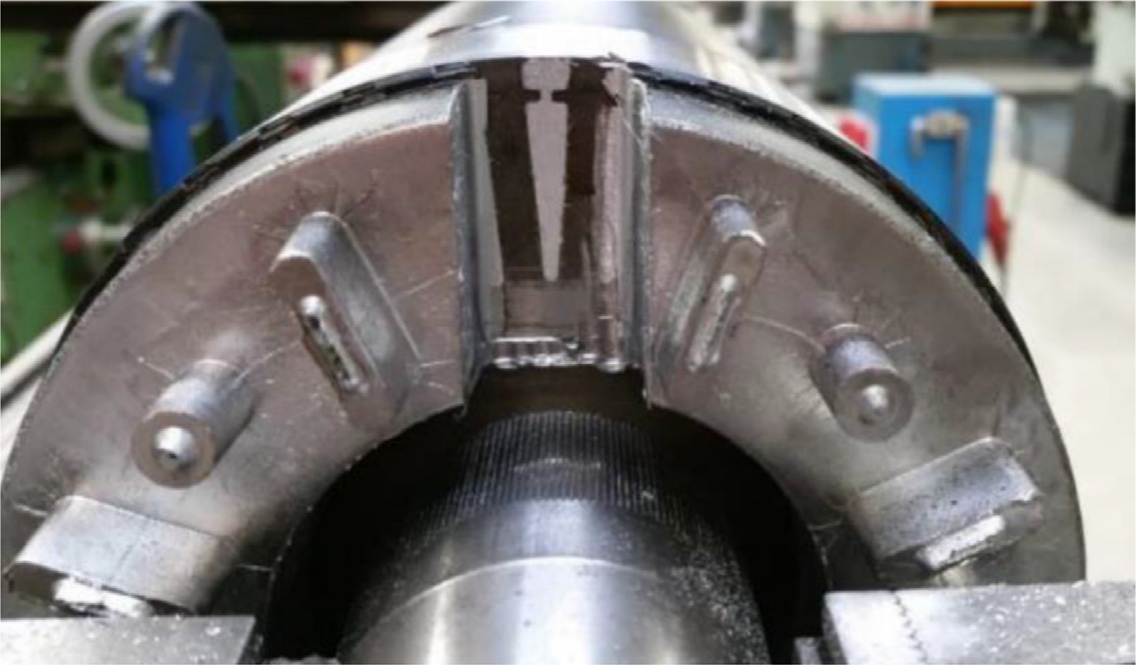
Broken rotor bar simulation.

A broken rotor bar fault was obtained by modifying a new electric motor. The modification was carried out by Facta Uitgeest BV. The broken rotor bar was simulated by removing part of the rotor endplate, as seen in Fig. 6. The endplate is rebalanced afterwards to prevent the unbalance to dominate the measurements. It must be noted that there is no normal/healthy behaviour measurement of this specific motor, due to the way the damage was simulated.

#### 3.3.4 Bent shaft

**Fig. 7:**
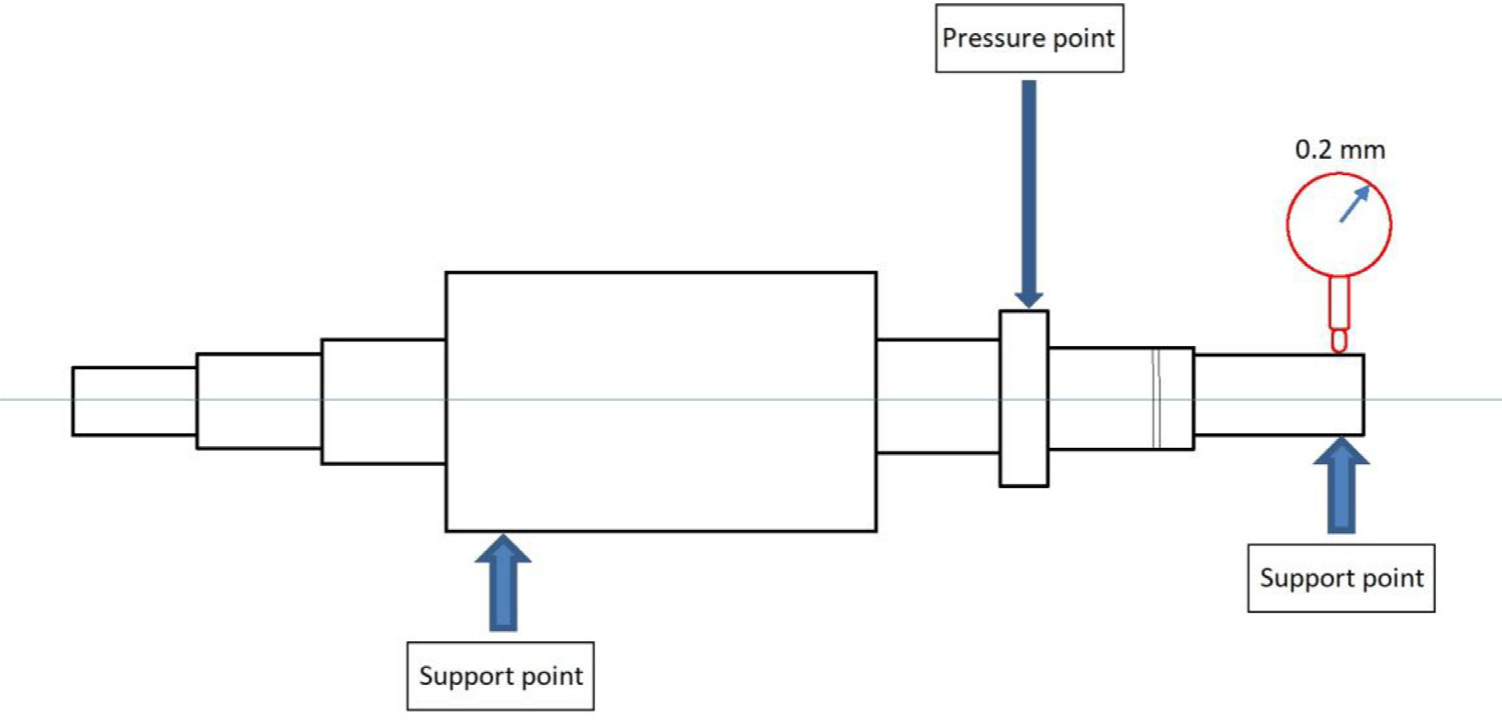
Schematic for bending of the shaft.

The shaft of a new electric motor was altered to implement a bent shaft fault. The modification was carried out by Facta Uitgeest BV. The entire rotor was loaded by a three-point bending press, resulting in a permanent eccentricity of 0.2 mm. The schematic of the procedure is presented in Fig. 7. It must be noted that there is no normal/healthy behaviour measurement of this specific motor, due to the way the damage was introduced.

#### 3.3.5 E-motor bearing damage

Bearing damage was simulated in 3 different ways:

(i) raceway damage
(ii) roll element damage
(iii) contamination of the grease

**Fig. 8:**
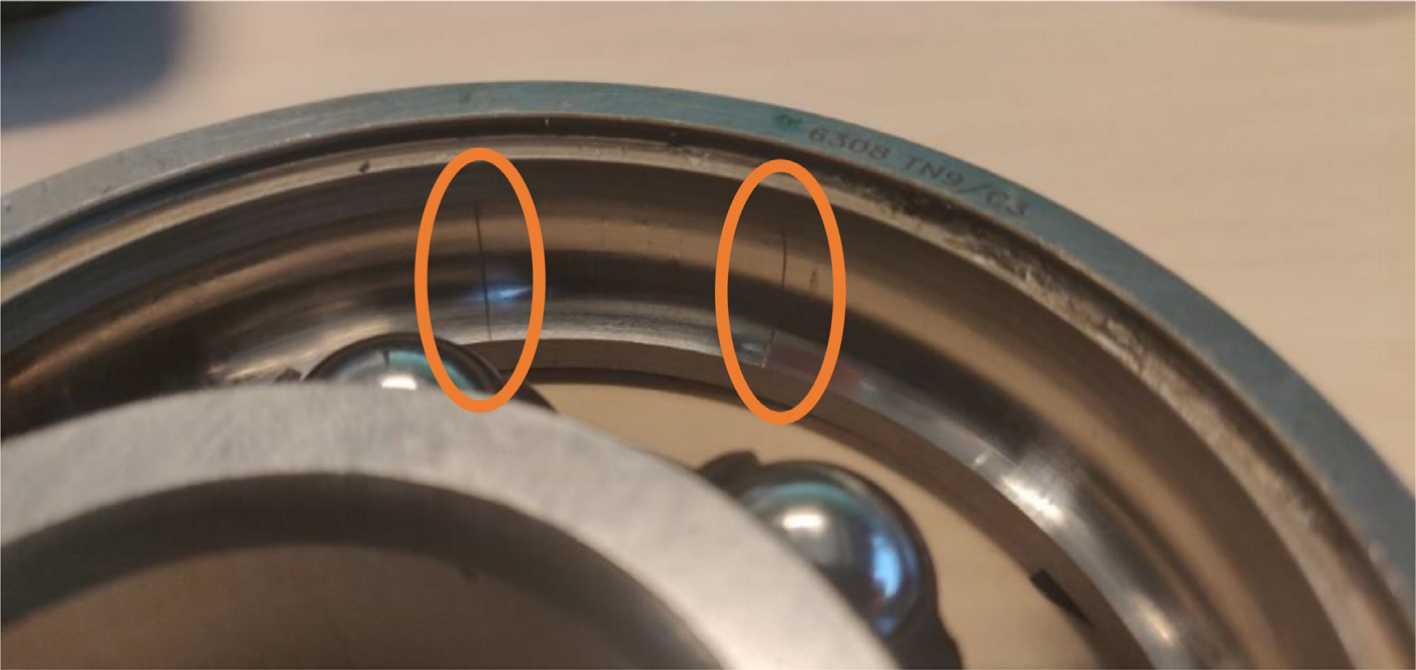
BPFO bearing damage severity 2.

Raceway damage is split in two individual damage cases: inner and outer ring damage. Either inner or outer ring damage is applied, since only one single damage is used per case. The raceway damages are referred to as “BPFI” and BPFO”, due to their effect on the ball pass frequency. The modified bearings were supplied by SKF Houten BV. A uniform line was milled in the raceway in axial direction. The dimensions of the line are 1 mm wide and 350 µm deep. To simulate a higher severity, 1 and 2 parallel lines were added to the first line for severity 2 and 3 respectively. The additional lines are located at 70% of the distance between the rolling elements. Severity level 2 of the BPFO is shown for reference in Fig. 8.

**Fig. 9:**
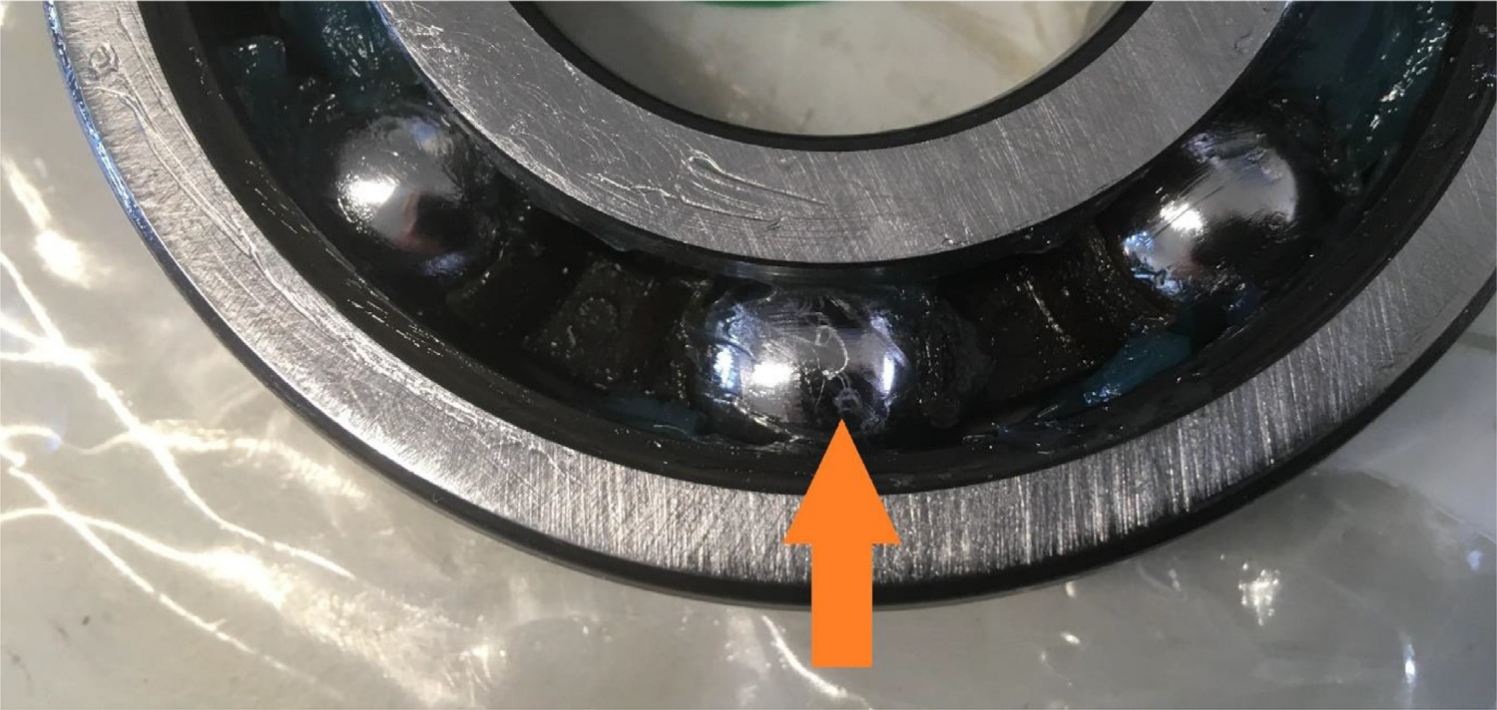
Damaged rolling element.

The rolling element damage (affecting the Ball Spin Frequency, BSF) was generated by irregularly manipulating one of the rolling elements with a rotary tool. The rolling element was damaged all around to prevent a situation where the damage does not make contact with the raceway, see Fig. 9.

Finally, the lubricant contamination was created by mixing in iron filings with the bearing's grease. By letting the motor run for a while before performing a measurement, the filings are expected to be well distributed throughout the bearing. All bearing damage simulations for the electric motor have been applied on the non-driven end bearing of the motor. The corresponding bearing damage frequency factors, defined as multiplication factor of the rotational frequency are listed in Table 5.

**Table 5:** Relative bearing damage frequencies as multiplication factor of rotational frequencies [12].

6308 (pump)			6309 (motor)		
BPFO	BPFI	BSF	BPFO	BPFI	BSF
3.072	4.928	4.078	3.037	4.963	3.911

#### 3.3.6 Pump bearing damage

**Fig. 10:**
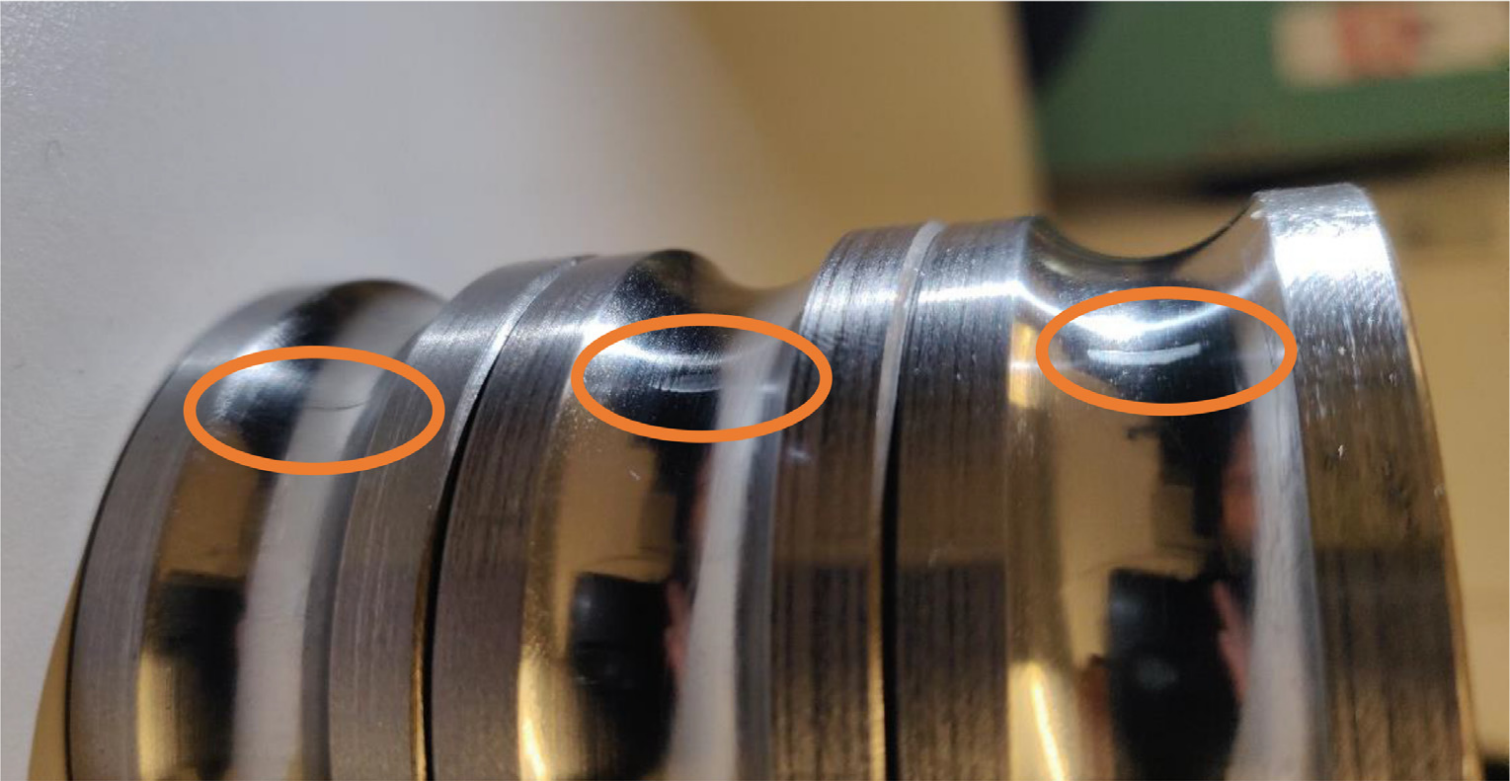
Unintentional assembly damage in inner ring 6308.

The pump bearing damage in the outer ring was machined in the same manner as specified above. The damage was introduced in the bearing at the non-driven end of the pump. However, during assembly of the bearing, another damage occurred in the inner ring of this bearing by accident. This mistake was consistent for every severity level of the pump bearing damage, see Fig. 10.

#### 3.3.7 Impeller damage

**Fig. 11:**
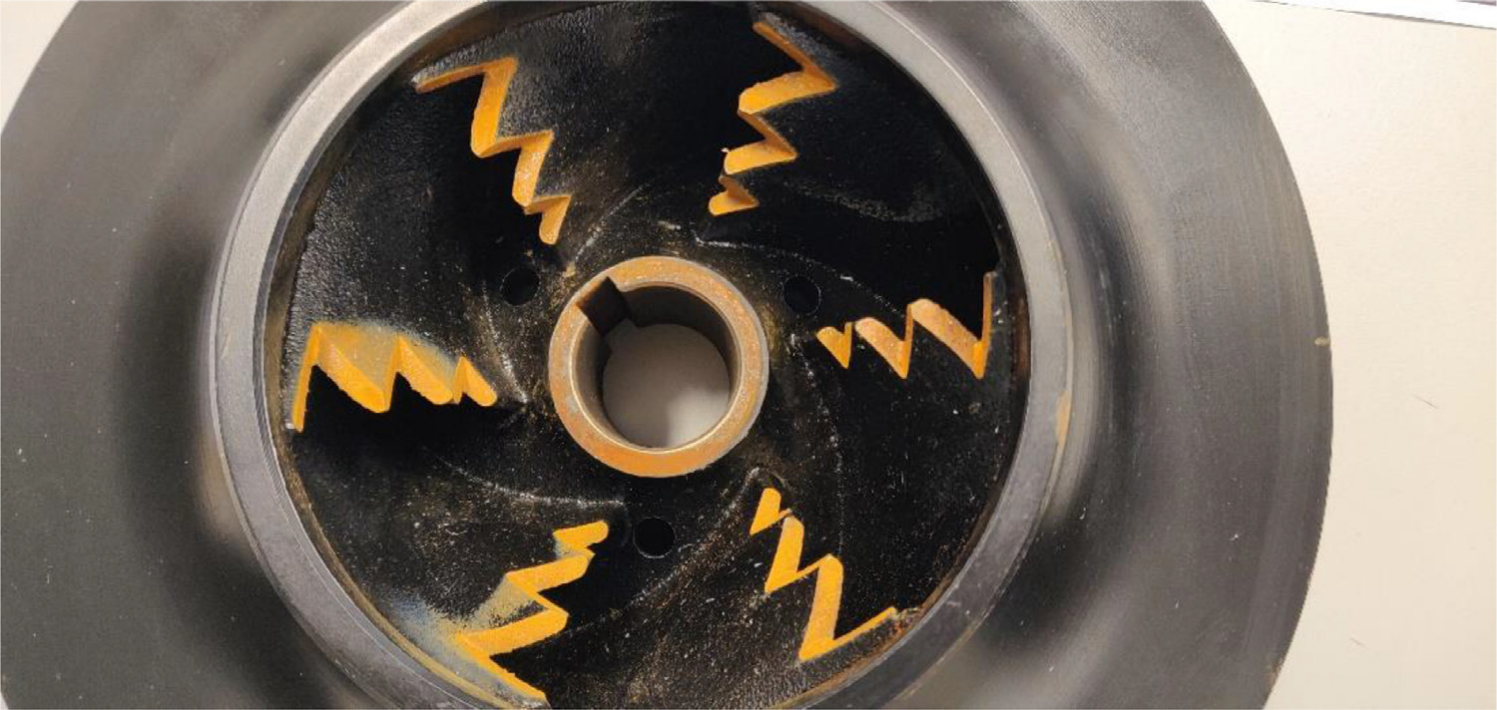
Impeller damage 3 at suction side.

**Fig. 12:**
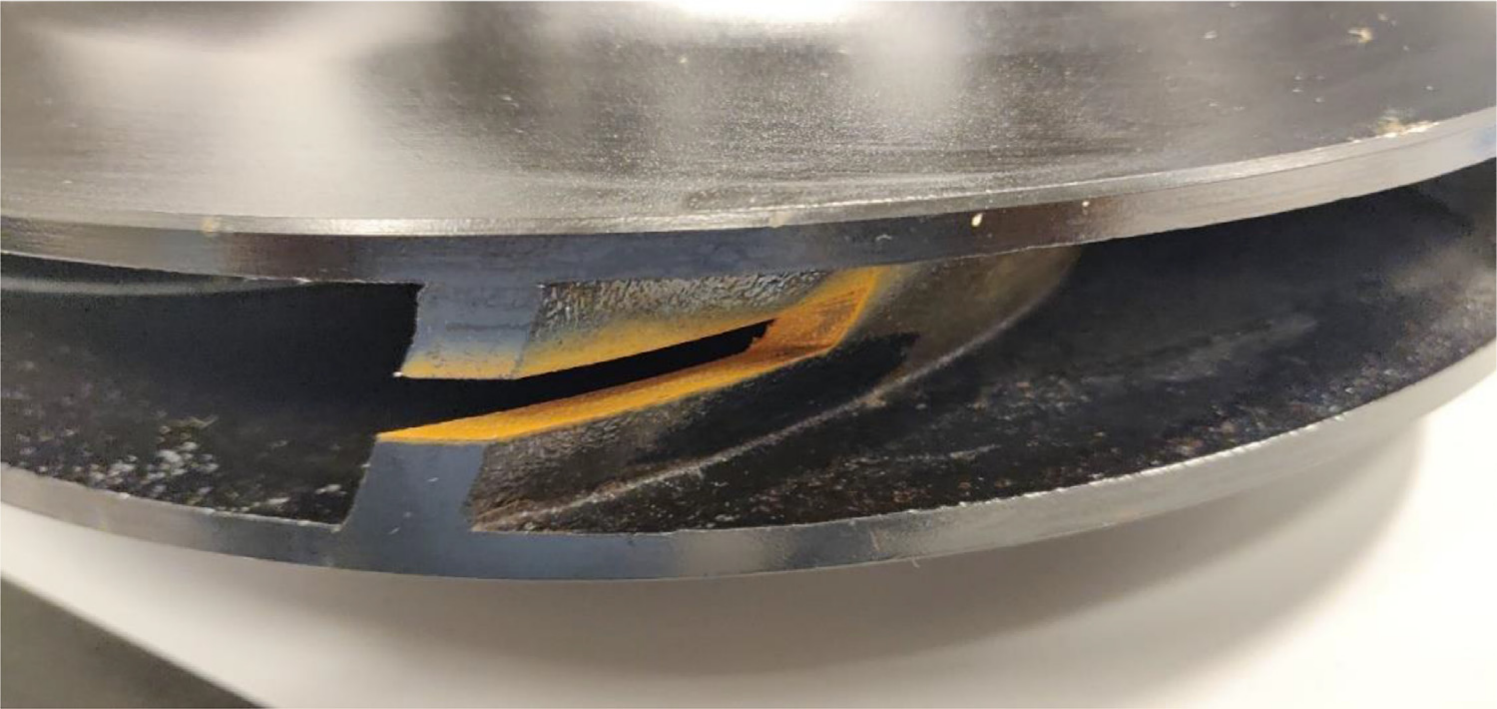
Impeller damage 3 at discharge side.

The impeller was damaged by milling away specific sections from the impeller blades. This simulates damage as a result of cavitation. In real world scenarios, this damage can be very evenly distributed over the impeller, resulting in very little unbalance in the impeller. To prevent unbalance from becoming a factor in the simulation, the impellers were rebalanced after the milling process. The corresponding balancing reports are also available in the dataset. The different severities are created by milling away more material than for the previous severity. The manner in which the damage was applied can be viewed in Fig. 11 for the suction side and Fig. 12 for the discharge side.

#### 3.3.8 Reduced flow and cavitation

The set-up contains a valve on the suction side of the pump and a valve on the discharge side of the pump. By manipulating these, and thus reducing the magnitude of the flow through the pump, occurrence of cavitation inside the pump can be mimicked. It must be noted however, that the pump did not run completely smooth in the initial set-up (with unrestricted flow) and therefore cavitation was not completely absent in the healthy case. This means that the cavitation in this dataset cannot be considered to be a completely realistic example. However, during the experiment the magnitude of the flow was changed, which alters the operating state of the machine. This deviation can be noted in the measurements. The different severities of reduced flow were created by closing the valves in several steps. The steps were based on the loss in flow measured by the flow gauge. The inlet valve was never completely closed to prevent collateral damage in the pump. The outlet valve was completely closed in the most severe case. The approximate flow per severity is shown in Table 6.

**Table 6:** Cavitation severities based on different reductions of flow.

Flow(m^3^/h)	Healthy	Severity 1	Severity 2	Severity 3	Severity 4	Severity 5
Suction	125	105	85	55	25	-N/A
Discharge	125	90	75	50	20	0

#### 3.3.9 Misalignment

**Fig. 13:**
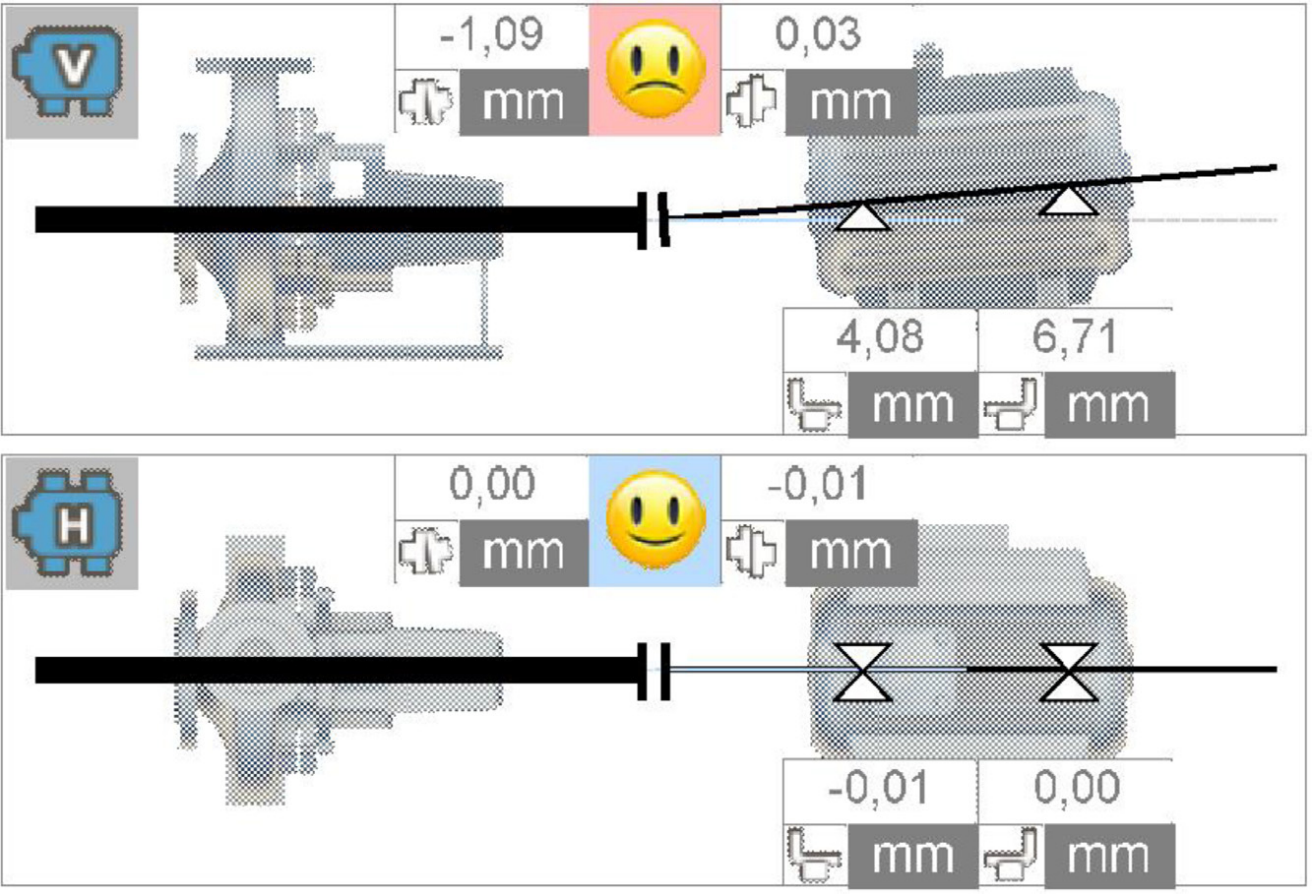
Extract of angular misalignment 1.1 mm.

Misalignment was achieved by adding shims under the motor's feet. By adding the same amount under each foot, a parallel misalignment can be achieved. Altering the amount of shims under the feet on the non-driven side with respect to the amount of shims under feet on the driven side allows for the creation of pure angular misalignment, or a combination of parallel and angular misalignment. The number of shims can be gradually increased to realise different severities. For every implemented severity, the actual alignment is quantified and documented in the alignment reports. In Table 7 the severity numbers with their corresponding misalignments are presented. For angular misalignment, the angle is denoted in millimetre deflection over a 100 mm distance, an example of this is presented in Fig. 13. The parallel misalignment is presented in millimetre deflection from the perfect alignment in vertical direction. The combination misalignment value represents a combination of the angular and parallel deflection.

**Table 7:** Misalignment severities (in mm).

	Severity 1	Severity 2	Severity 3	Severity 4	Severity 5
Angular	0.1	0.2	0.3	0.5	1.1
Parallel	0.1	0.2	0.5	1.0	N/A
Combination	0.1 + 0.1	0.2 + 0.2	0.5 + 0.5	1.0 + 1.0	N/A

#### 3.3.10 Unbalance

**Fig. 14:**
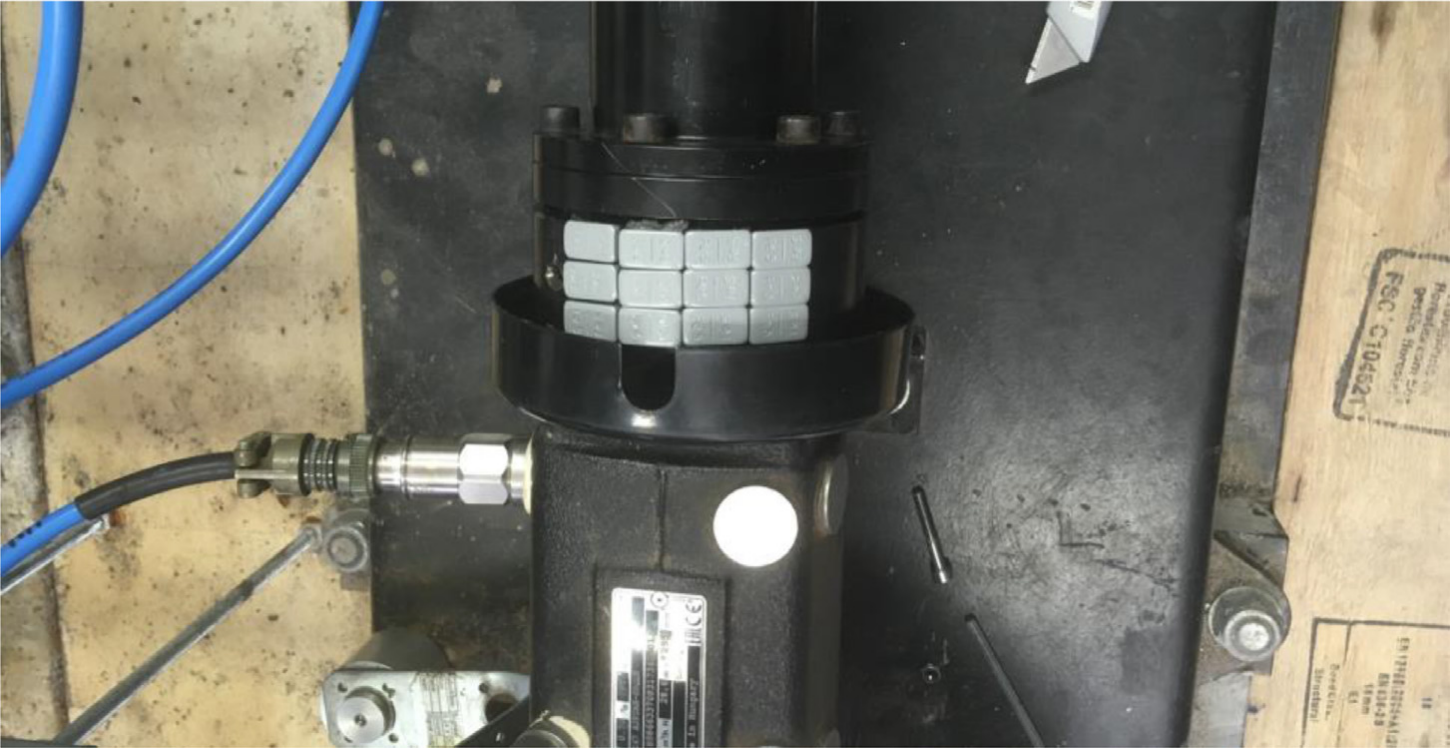
Added weights to create unbalance at the pump side (60 g).

Unbalance was simulated by attaching wheel weights to the coupling. Since the coupling exists of two parts, the wheel weights are attached to either the electric motor's side of the coupling, or the pump's side of the coupling. An example of the weights added to the pump side of the coupling is presented in Fig. 14. The weights were tightly accumulated on one side of the shaft, to avoid counteracting the impact of the increased weight due to the shift of the resulting centre of mass towards the centre of rotation. The different levels of severity were implemented by adding more weights, as shown in Table 8.

**Table 8:** Unbalance severities.

	Severity 1	Severity 2	Severity 3	Severity 4	Severity 5	Severity 6
Motor side	5 g	10 g	20 g	30 g	80 g	120 g
Pump side	30 g	60 g	90 g	N/A	N/A	N/A

#### 3.3.11 Coupling degradation

**Fig. 15:**
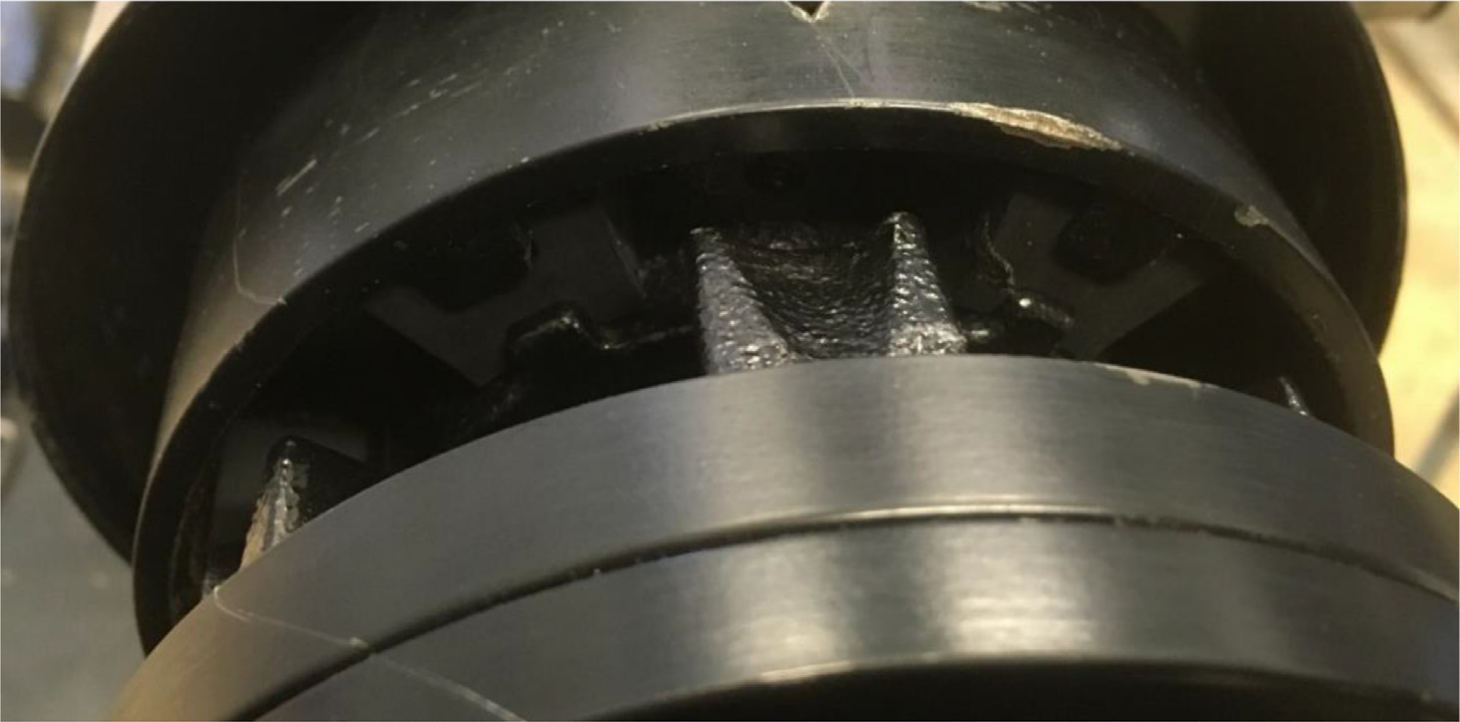
Claw coupling with rubber H-Blocks.

**Fig. 16:**
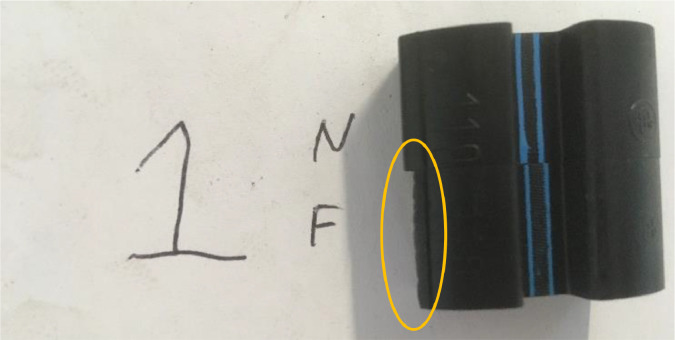
Case 1 of coupling damage.

The coupling consists of two main sections, the pump side and the motor side. These are claw shaped sections that interlock to form a coupling, see Fig. 15 for a close-up view. Six rubber H-blocks are fitted between the prongs of the claws to prevent metal to metal contact. These rubber blocks usually degrade over time, and this degradation can be accelerated by frequently starting and stopping or due to misalignment. This degradation was simulated by cutting away some material on the outside of the blocks, where it would touch the claw. This is done at different levels. By cutting away more material on one side, a more severe case is created. This is denoted as case 1 for the smallest severity and case 2 for the highest severity. Case 1 is displayed in Fig. 16, where N refers to the new block and F to the simulated failed block. Another approach was to cut away material off of both sides of the blocks, this is denoted as case 2D. The final case was made by replacing one of the six most severely damaged blocks by one of the least damaged blocks, which is denoted as case 3. This simulates a situation where only one H-block is replaced as a maintenance action, instead of the whole set.

#### 3.3.12 Loose foot

**Fig. 17:**
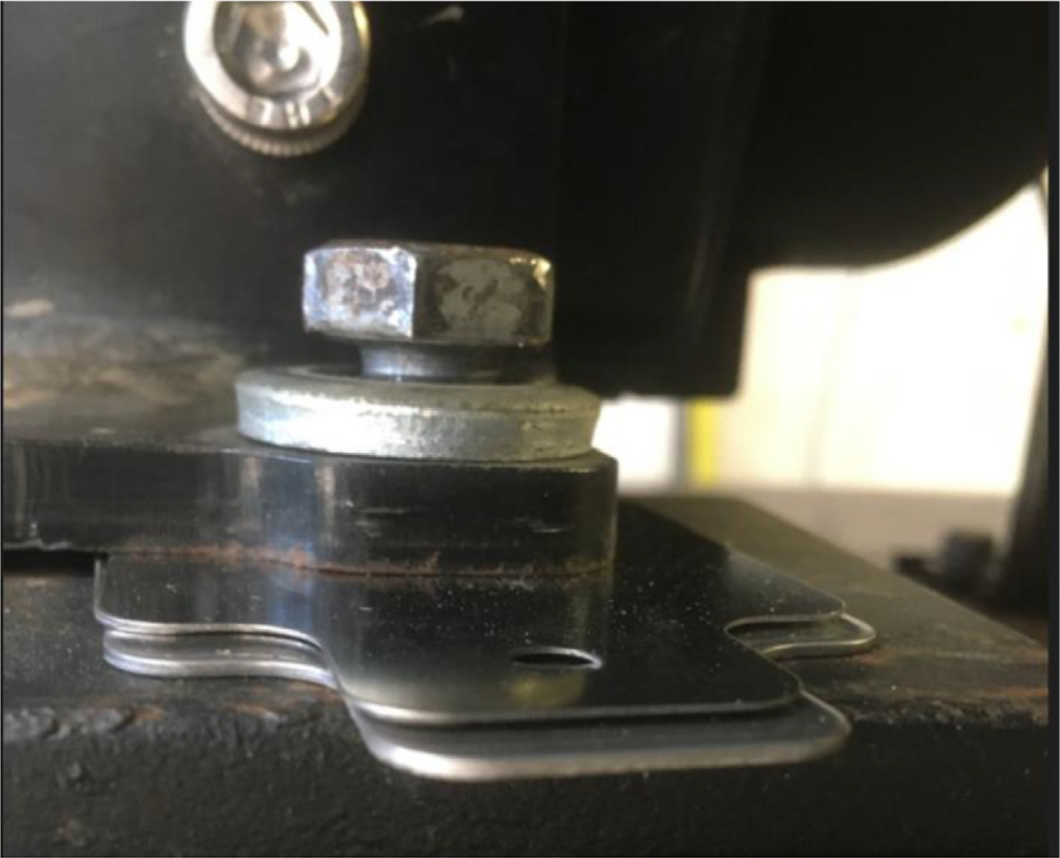
loosened bolt right side driven end electric motor.

For the loose foot failure, one of the bolts keeping down the electric motor or pump was released. In case of the motor, the right foot on the driven-end is loosened, such that the bolt head is not in contact with the motor foot, see Fig. 17. In case of the pump, the left driven-end bolt is loosened to the same degree. It must be noted that this also influenced the alignment (see the corresponding alignment report for details).

#### 3.3.13 Soft foot

**Fig. 18:**
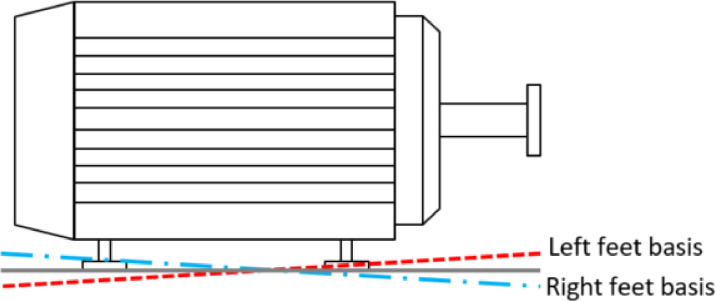
Soft foot schematic.

Soft foot is a phenomenon where the stator housing of the electric motor is twisted around the shafts axis. The result is an eccentric air gap between the rotor and the stator. This was simulated by placing more shims underneath two diagonally opposing feet than underneath the other two diagonally opposing feet. Schematically, this is visualised in Fig. 18. This creates a torsional deflection of the stator housing. It must be noted that this also alters the alignment. The first severity is created with 0.5 mm shims underneath the driven-end left and non-driven-end right motor feet. The second severity is created with 1.0 mm shims underneath the same motor feet.

#### 3.3.14 Noise

Another setting that influences vibration parameters is when surrounding machines are running as well. To achieve this, all four electric motor pomp set-ups present in the test facility were activated. Motor 1 was set to 65% of rated speed whilst motor 3 was set to 100 % rated speed. Motor-pump set-up 2 and 4 were both measured during this operation, the measurements of motor 4 were stored only whilst motor 2 was running at 100%. This created a noisy environment, but with healthy running machines.

## Limitations

Due to the restricted time available for using the test facility and a finite project budget, the data collection has resulted in some limitations in the dataset. Firstly, the number of measurements per fault is limited and the faults were not duplicated on different setups. Further, due to automated storing of the data at specific times, some measurements in the dataset may (partially) contain data of non-running equipment. Some measurements turned out to have corrupted data, the folders exist, but are empty. Another limitation is that the two measurement techniques were not synchronised, which means that the measurements for a specific fault are not time-synchronized and operating conditions might slightly vary.

As new electric motors had to be modified for the broken rotor bar and bent shaft faults, data for the undamaged situation on exactly the same motor is not available, but has to be obtained from a similar healthy motor. This means, however, that not every difference in measurements can be purely attributed to the simulated problem. Electric motors have inherent differences as a result of the production process, which alters the dynamic response compared to other motors of the same type.

A final limitation in the set-up was that cavitation was never completely eliminated, due to the imperfect piping layout.

## Ethics Statement

The authors have read and follow the ethical requirements for publication in Data in Brief and confirm that the current work does not involve human subjects, animal experiments, or any data collected from social media platforms.

## CRediT authorship contribution statement

**S. Bruinsma:** Conceptualization, Methodology, Investigation, Data curation, Writing – original draft. **R.D. Geertsma:** Writing – review & editing, Supervision. **R. Loendersloot:** Writing – review & editing, Supervision. **T. Tinga:** Writing – review & editing, Supervision.

## Data Availability

Motor Current and Vibration Monitoring Dataset for various Faults in an E-motor-driven Centrifugal Pump (Original data) (4TU.ResearchData).
